# Impact of an Electronic Alert in Combination with a Care Bundle on the Outcomes of Acute Kidney Injury

**DOI:** 10.3390/diagnostics12123121

**Published:** 2022-12-10

**Authors:** Ana Carolina Nakamura Tome, Rodrigo José Ramalho, Karise Fernandes dos Santos, Bianca Ponte, Helga Agostinho, Mauricio Nassau Machado, Marcelo Barreto Lopes, Mario Abbud-Filho, Emerson Quintino de Lima

**Affiliations:** 1 Hospital de Base, Sao Jose do Rio Preto, Sao Paulo 15090-000, Brazil; 2 Division of Nephrology, Sao Jose do Rio Preto Medical School, Sao Jose do Rio Preto, Sao Paulo 15090-000, Brazil; 3 D’Or Institute for Research and Education (IDOR), Nephrology Department at Hospital Sao Rafael, Salvador 41253-190, Brazil

**Keywords:** acute kidney injury, electronic alert, care bundle, multidisciplinary education, clinical pharmacist

## Abstract

Early diagnosis is essential for the appropriate management of acute kidney injury (AKI). We evaluated the impact of an electronic AKI alert together with a care bundle on the progression and mortality of AKI. This was a single-center prospective study that included AKI patients aged ≥ 18 years, whereas those in palliative care, nephrology, and transplantation departments were excluded. An AKI alert was issued in electronic medical records and a care bundle was suggested. A series of classes were administered to the multidisciplinary teams by nephrologists, and a clinical pharmacist audited prescriptions. Patients were categorized into pre-alert and post-alert groups. The baseline characteristics were comparable between the pre-alert (n = 1613) and post-alert (n = 1561) groups. The 30-day mortality rate was 33.6% in the entire cohort and was lower in the post-alert group (30.5% vs. 36.7%; *p* < 0.001). Age, pulmonary disease, malignancy, and ICU admission were associated with an increase in 30-day mortality. The electronic AKI alert together with a care bundle and a multidisciplinary education program was associated with a reduction in 30-day mortality in patients with AKI.

## 1. Introduction

Acute kidney injury (AKI) affects all age groups, regardless of sex, ethnicity, or social class, and can present in community or hospital settings. Despite the marked reduction in AKI-associated mortality observed in recent decades, its incidence remains very high, reaching up to 22% of all hospitalized patients [1]. Several studies demonstrate that AKI is an independent risk factor for death; the mortality rate rises with increasing AKI severity, reaching rates of 50–60% in patients requiring renal replacement therapy [1,2,3]. Progression to chronic kidney disease (CKD), which can be predicted based on the severity and duration of AKI, can induce an increase in the number of patients with end-stage renal disease and the need for specialized follow-up [4]. In addition, AKI is associated with higher costs and longer hospital stays [2]. Therefore, it is essential that health professionals be aware of its risk factors so they can recognize them and respond in an early manner, as most patients are not managed by nephrologists [5,6]. A review by the National Clinical Enquiry into Patients Outcomes and Death focusing on the care of patients who died with AKI at hospitals in England has revealed that only half of the patients were managed well, with AKI recognized late in 43% of the patients and predictable and preventable AKI being observed in one-fifth of these cases [7]. In response, the International Society of Nephrology has implemented the 0by25 initiative to reduce preventable AKI deaths to zero by 2025 [8].

Alerts in electronic medical record (EMR) systems are a strategy for early AKI detection, and the combination of such alerts with care bundles can provide an opportunity for the further education of healthcare professionals and an improvement in patient care [9,10,11,12]. We aimed to determine the impact of implementing an electronic alert system in combination with an institutional care bundle and educational program on patients with AKI in a general teaching hospital.

## 2. Materials and Methods

### 2.1. Setting and Participants

This prospective cohort study was conducted at Hospital de Base, Sao Jose do Rio Preto Medical School, Brazil, between 1 January 2018 and 31 December 2018. This institution is a teaching hospital and tertiary care center for all specialties, serving a regional population of 2,000,000, with 966 beds, including 217 intensive care unit (ICU) beds.

The inclusion criterion for the present study was patients aged ≥18 years. The exclusion criteria were patients admitted to the nephrology department and palliative care wards with end-stage renal disease or basal serum creatinine levels of >4.0 mg/dL.

### 2.2. Electronic Alert and Care Bundle

An electronic AKI alert, which was created using the EMR system, analyzed patient serum creatinine values in real time using the 2012 Kidney Disease: Improving Global Outcomes (KDIGO) criteria for AKI (an increase of ≥0.3 mg/dL within 48 h or a ≥1.5 times increase from the baseline within seven days) [13] (Appendix A). Urine output was not used as an AKI criteria due to the difficulties in obtaining urine volume in the wards.

Baseline serum creatinine was defined as the lowest value within the six months prior to admission. In patients without available pre-admission values, the first serum creatinine value after admission was included. Estimated glomerular filtration rate (eGFR) was calculated using the Chronic Kidney Disease Epidemiology Collaboration (CKD-EPI) formula [14].

After the diagnosis of AKI, a non-interruptive alert displaying “Warning: probable acute kidney injury” and a link to a care bundle were issued in the patient’s EMR, which can be accessed by the medical and nursing staff. The care bundle (Appendix B) was based on the KDIGO recommendations [15] and the London AKI app [16] and was advertised in the form of banners, posters, and videos. Training sessions on electronic alerts (how, when, and with whom it would work) and AKI (diagnosis, classification, etiology, management suggestions, and when to call a nephrologist for consultation) were provided to the multidisciplinary teams by the AKI physicians for 1 month.

A clinical pharmacist reviewed all prescriptions issued for patients with KDIGO stage III AKI every day and those for patients with KDIGO stage I or II every 48 h. If necessary, attending physicians were notified by telephone of recommendations for prescription changes, such as drug dose adjustment, withdrawal of nephrotoxic drugs, drug interactions, and reductions in infusion volumes.

### 2.3. Data Collection

The electronic alert system and care bundle program was initiated on 10 July 2018. According to the data collected from patients with AKI in previous years, our service is not affected by seasonality in relation to incidence or mortality. Therefore, the post-alert group included eligible patients who were treated using the electronic alert system and care bundle between 10 July 2018 and 31 December 2018, and the pre-alert group comprised patients treated between 1 January 2018 and 9 July 2018, when the AKI electronic alert ran in the background and was not being reported to clinicians. Before the initiation of the electronic alert, for a period of three months, the data collected from the system were audited by a nephrologist.

Comorbidities were grouped according to medical specialty and were collected by an EMR review as reported by the attending physician. Coronary artery disease, peripheral arterial disease, hypertension, chronic heart failure, and cardiac arrhythmia were considered cardiovascular diseases; chronic obstructive pulmonary disease and asthma as pulmonary diseases; liver cirrhosis and peptic ulcers as gastrointestinal diseases; ischemic strokes and dementia as neurologic diseases; and diabetes and obesity as endocrine diseases. Rheumatologic diseases were reported for those who had an autoimmune condition. Malignancy included solid tumor and hematologic malignancy. Acquired immunodeficiency syndrome and the use of immunosuppressive drugs were considered immunosuppressive conditions.

Only the first hospitalization during which the patient developed AKI was considered for data collection. AKI progression was assessed by analyzing the number of patients who were diagnosed as presenting with KDIGO I or II stages that progressed to worse levels. The current study evaluated mortality during the first 30 days after hospitalization. Adherence to the AKI care bundle was assessed 24 h before and after the EMR alert.

### 2.4. Statistical Analysis

Parametric data are presented as means ± standard deviation, and non-parametric data are presented as medians with interquartile ranges. Continuous variables were compared using the non-parametric Mann–Whitney test for independent samples. The chi-square or Fisher’s exact test was used to compare independent categorical variables, and McNemar’s test was used for paired data. The Cox univariate and multivariate proportional hazard models were used to determine the association between AKI and 30-day mortality. The Benjamini–Hochberg procedure was used to estimate *p*-values corrected for multiple comparisons [17]. The following variables were included in the multivariate model: age (<40 years, 40 to <65 years, 65 to <75 years, and ≥75 years), ICU admission, baseline eGFR (each 10 mL/min/1.73 m^2^ increment), pulmonary disease, neurologic disease, malignancy, immunosuppressive conditions, and electronic AKI alert. *p*-values < 0.05 were considered to indicate statistical significance.

## 3. Results

**Figure 1:**
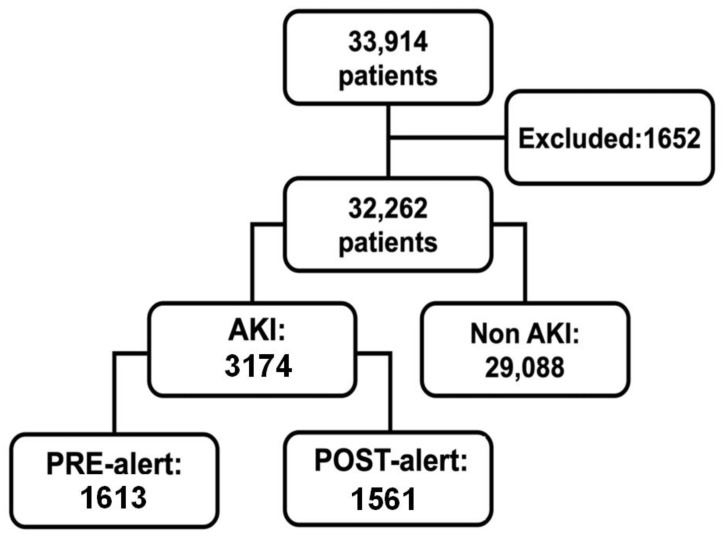
Flowchart of the study population showing the categorization of the 33,914 patients hospitalized in the year 2018. A total of 1652 patients were excluded from this study, and 3174 patients developed AKI; 1613 in the pre-alert period and 1561 in the post-alert period.

In 2018, 33,914 patients were hospitalized, 1652 of whom were excluded based on the exclusion criteria (Figure 1).

A total of 3174 patients were diagnosed with AKI between 1 January 2018 and 31 December 2018, representing 9.8% of all hospitalizations. Of these, there were 1613 and 1561 patients in the pre-alert and post-alert groups, respectively, and the AKI incidence was similar between the two groups (10.2% and 9.5% in the pre-alert and post-alert groups, respectively; *p* = 0.19). Furthermore, AKI occurred across all specialties and the AKI incidence by department was as follows: internal medicine (11.7%), cardiology (11.6%), neurosurgery (10.7%), orthopedy (8.3%), general surgery (8.2%), and hematology (7.0%).

### 3.1. Baseline Characteristics

As presented in Table 1, baseline characteristics were similar between the pre-alert and post-alert groups. The overall cohort comprised 1839 (57.9%) male patients, and the mean age was 66 years. Additionally, 88.5% of the patients self-declared as white and 65.6% of the patients were in the ICU at the time of AKI diagnosis. The baseline serum creatinine and eGFR values were 1.1 (0.8–1.5) mg/dL and 65 (41–92) mL/min/1.73 m^2^, respectively. The AKI diagnosis was based on an increase of ≥0.3 mg/dL in serum creatinine within 48 h for 68.7% of the cases.

Regarding comorbidities, the pre-alert group had more patients with immunosuppression and gastrointestinal disease.

### 3.2. AKI Severity

At the time of AKI diagnosis, the rates of patients with KDIGO stage I, II, and III were comparable between the pre-alert and post-alert groups (Table 2). The number of patients who remained in AKI stage 1 in the post-alert group was higher than in the pre-alert group.

### 3.3. Nephrology Consultation and Renal Replacement Therapy

The number of nephrology consultation requests and the time delay were similar between the pre-alert and post-alert groups (Table 2). Renal replacement therapy was performed on 514 (15%) patients, and no significant difference was observed between the pre-alert and post-alert groups (14.9% and 15.1%, respectively; *p* = 0.929). Similarly, the length of hospital stay did not differ between the pre-alert and post-alert groups.

### 3.4. Care Bundle Adherence

After the AKI alert, fewer hypotension episodes (mean arterial pressure < 65 mmHg or mean systolic blood pressure < 90 mmHg) were observed. Similarly, there was a decrease in the use of nonsteroidal anti-inflammatory drugs (NSAIDs), angiotensin-converting enzyme inhibitors (ACEis) and angiotensin receptor blockers (ARBs). The number of blood glucose tests performed during the first 24 h after the alert was higher, and there were more hyperglycemia episodes in the post-alert group. A new serum creatinine test was performed in the first 24 h of the AKI electronic alert in only 43% of the patients (Table 3).

### 3.5. Mortality

**Figure 2:**
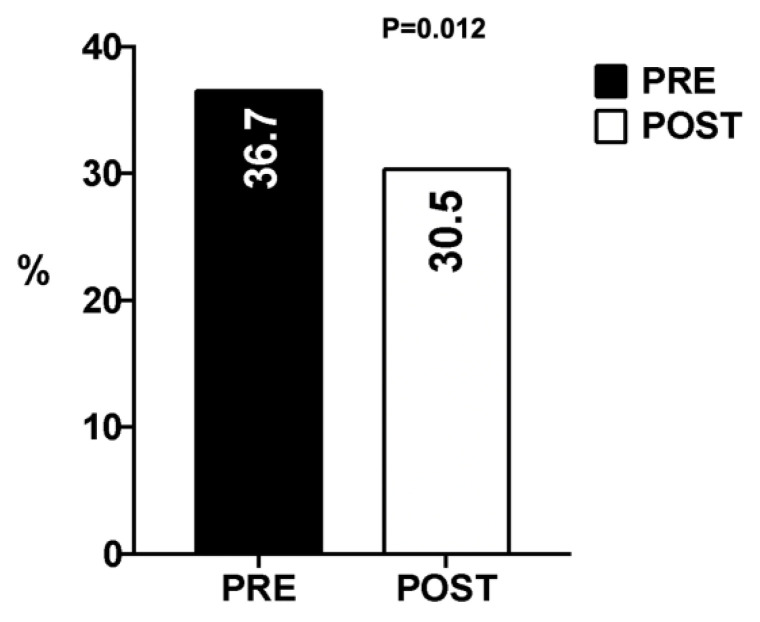
Acute kidney injury 30-day mortality rates.

**Figure 3:**
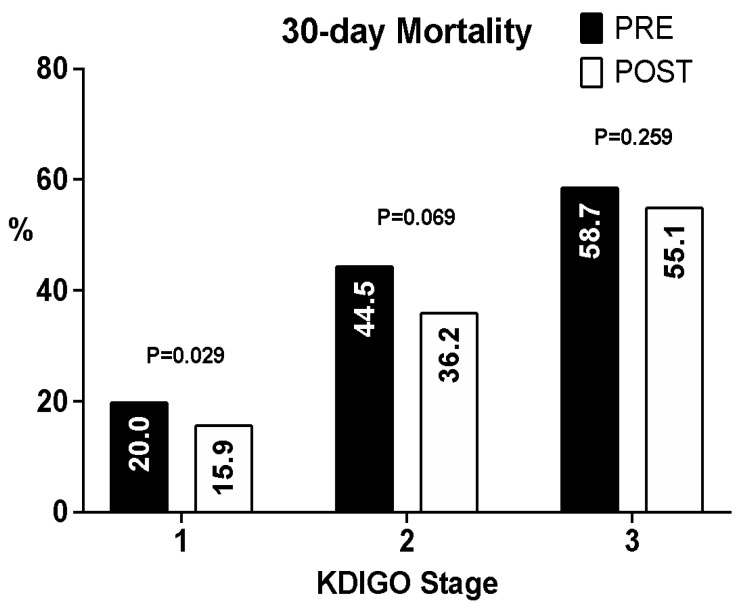
Acute kidney injury 30-day mortality according to the KDIGO stage.

The 30-day mortality was 33.6% for the whole cohort and was lower in the post-alert group than in the pre-alert group (30.5% vs. 36.7%, respectively; *p* = 0.012) (Figure 2). The assessment of mortality according to the KDIGO classification revealed that the 30-day mortality rates were lower for all KDIGO stages in the post-alert group than in the pre-alert group, although this reduction was statistically significant for patients with KDIGO stage I (Figure 3).

The analysis of independent predictive factors for 30-day mortality indicated that older age and ICU admission were associated with a higher risk of 30-day mortality. Other predictive factors were baseline eGFR, pulmonary disease, and malignancy. The AKI electronic alert in patients’ EMR was an independent predictive factor for 30-day mortality (Table 4).

## 4. Discussion

The development of AKI may be considered a parameter for hospital care quality [18]. Due to the persistently high incidence of AKI, significant efforts must be directed towards early diagnosis and interventions to reduce AKI mortality [8].

Despite the development of promising biomarkers for kidney injury, the clinical diagnosis of AKI continues to be based on an increase in serum creatinine and/or a reduction in urinary volume [19,20]. The integration of patient information and hospital processes has become possible with the increasingly widespread use of hospital management and EMR systems. Under the assumption that an early recognition of AKI could improve the condition of these patients and the quality of care they receive, an increasing number of studies on electronic alerts have been conducted in recent years, but the results of these are still uncertain [21]. We implemented a real-time electronic alert for AKI together with a care bundle and a multidisciplinary education program in a high-complexity hospital, with the aim of evaluating its impact on AKI.

Kashani et al. suggested implementing a proactive nephrology practice structure to reduce AKI incidence. The authors proposed the creation of a team of nephrologists to monitor all hospitalized patients to manage risk factors before AKI development [22]. Goldstein et al. reported that reducing exposure to nephrotoxic drugs diminished AKI incidence in pediatric patients [23,24]. In both studies, modification of risk factors for the development of AKI was performed, reducing the incidence of AKI. In our study, patients were alerted when AKI wasdiagnosed. These findings highlight the fact that nephrologist intervention should possibly be conducted before the development of AKI to manage the risk factors and decrease AKI incidence.

In the present study, a higher rate of patients remained in AKI stage 1 in the post-alert group compared with the pre-alert group. In contrast to the study conducted by Park et al. [25], in which the authors observed the beneficial impact of an electronic alert with automated nephrologist consultation and demonstrated that earlier nephrologist involvement improved AKI recovery, in our study, the consultation was carried out as required by the attending physician. Early nephrologist consultation has already been shown to reduce AKI progression, length of hospital stay, and mortality [26,27,28].

Even with AKI management and the implementation of an education program, there is still a need for improvement in the care process. The alert used did not impede any of the prescriptions issued by the clinicians. Faster responses from physicians were observed in studies that assessed interruptive alerts [29]. In the present study, less than half of the patients had a new creatinine test performed in the first 24 h after the alert, possibly because the physician did not access the EMR in time to request and perform a new creatinine test. Barker et al. demonstrated an increase in creatinine requests in primary and secondary care services in the United Kingdom. However, the alert was implemented one year after the intervention (training of the multidisciplinary team) [30]. To the best of our knowledge, no study has evaluated creatinine requests for AKI patients after implementing an electronic alert.

Although fluid balance was recommended in our care bundle, the rate of patients with a complete fluid chart 24 h before and after the electronic alert was similar. The same difficulty in obtaining data on urine output and fluid balance was reported in a study that attempted to improve AKI management with a care bundle and audit cycles [31].

The reduced number of hypotensive episodes within the first 24 h after the electronic alert in the present study was comparable to that reported by Colpaert et al., who observed that a higher percentage of patients received fluids or vasopressors 60 min after the AKI alert [32]. The lower number of hypotensive episodes may be one of the main factors that contributed to decreased mortality in our study.

In spite of the reduction in the rate of NSAIDs prescribed after the alert, the rate of patients using it remained significant. A decrease in the use of ACEis/ARBs was also observed. Although the impact of renin–angiotensin system inhibition on the clinical outcomes of patients with AKI remains controversial, its use is usually temporarily removed in clinical practice [33,34]. Al-Jaghbeer et al., in a study between 2012 and 2015 comparing pre- and post-alert groups, also evaluated the suspension of nephrotoxic drugs (NSAIDs, ACEI/ARB, aminoglycosides, contrast, and vancomycin) and found a reduction only in the use of contrast in the group of alerted patients. Even so, the analysis showed a decrease in mortality, length of stay, and need for renal replacement therapy, suggesting that there may have been other changes in the care of these patients that were not measured [35].

Although the benefits of electronic AKI alerts are promising, the results of these interventions remain conflicting [36,37]. Wilson et al. conducted a randomized study in a teaching hospital to evaluate the utility of an automated alert and a link to a website containing information on KDIGO recommendation guidelines, and reported that there were no improvements in AKI progression, the need for renal replacement therapy, or mortality rate [38]. Therefore, isolated electronic alerts may be inefficient. Similar to this study, other authors investigating the utility of integrated AKI care systems, comprising not only electronic alerts for early diagnosis but also care bundles and multidisciplinary teaching programs, have reported better outcomes [39,40,41]. In addition, mortality is known to be higher in the advanced stages of AKI. The higher number of patients who remained in AKI stage 1 found in our study may have contributed to the decrease in patient mortality in the post-alert group.

Recently, Wilson et al. conducted a multicenter randomized clinical trial in which the AKI electronic alert was implemented in six different hospitals in the USA and found distinct results and substantial heterogeneity of outcomes [42]. Our study is the first to be performed in South America in a tertiary teaching hospital, which may have local peculiarities that contributed to the results.

Though mortality was reduced in all stages, this reduction was statistically significant in patients with KDIGO stage I AKI. No published data distinguish mortality according to KDIGO stages. This finding might be further evidence that earlier diagnosis and a prompt response generate better outcomes.

The automation of the alert system is an advantage and was essential due to the large size of the institution and its high number of hospitalizations. In addition, the system is low-cost and reduces human error related to the inconsistency of the decision-making process.

### Limitation

The present study did not include data on the primary cause of hospitalization due to the design of the EMR system used, which does not use a diagnosis code. In addition, the AKI incidence in the present study might have been underestimated since the time elapsed before diagnosis by the algorithm did not include serum creatinine over the seven days prior to hospitalization, and patients with known AKI admitted with lower creatinine levels were not considered. Furthermore, unmeasured confounding factors may have contributed to the outcomes since the alert system was not randomized for patients.

## 5. Conclusions

The AKI 30-day mortality was reduced after the implementation of the electronic alert along with a care bundle and a multidisciplinary education program.

## Figures and Tables

**Table 1:** Baseline characteristics of patients with acute kidney injury.

	Overall	Pre-Alert	Post-Alert	*p*
	N = 3174 (100%)	N = 1613 (50.8%)	N = 1561 (49.2%)	
**Male**	1839 (57.9)	960 (59.5)	879 (56.3)	0.344
**White**	2808 (88.5)	1431 (88.7)	1377 (88.2)	0.728
**Age (years)**	66 (54–76)	66 (54–76)	66 (54–76)	0.728
18 to <40	297 (9.4)	155 (9.6)	142 (9.1)	0.728
40 to <65	1173 (37.0)	598 (37.1)	575 (36.8)	0.889
65 to <75	784 (24.7)	385 (23.9)	399 (25.6)	0.587
≥75	920 (29.0)	475 (29.4)	445 (28.5)	0.728
**Smoking**	673 (21.2)	355 (22.0)	318 (20.4)	0.587
**Alcoholism**	395 (12.4%)	193 (12.0)	202 (12.9)	0.718
**Body mass index (BMI)**	25 (22–28)	25 (22–28)	25 (23–29)	0.168
**Cardiovascular disease**	1907 (60.1)	960 (59.5)	947 (60.7)	0.728
**Pulmonary disease**	243 (7.7)	128 (7.9)	115 (7.4)	0.728
**Gastrointestinal disease**	289 (9.1)	172 (10.7)	117 (7.5)	0.024
**Neurologic disease**	476 (15.0)	246 (15.3)	230 (14.7)	0.728
**Rheumatologic disease**	81 (2.6)	47 (2.9)	34 (2.2)	0.576
**Malignancy**	519 (16.4)	282 (17.5)	237 (15.2)	0.344
**Immunosuppressive conditions**	135 (4.3)	90 (5.6)	45 (2.9)	0.022
**Endocrine disease**	1087 (34.2)	541 (33.5)	546 (35.0)	0.718
**ICU admission**	2082 (65.6)	1081 (67.0)	1001 (64.1)	0.344
**Baseline creatinine (mg/dL)**	1.1 (0.8–1.5)	1.1 (0.8–1.5)	1.1 (0.8–1.5)	0.587
**eGFR CKD-EPI (mL/min/1.73 m^2^)**	65 (41–92)	64 (41–91)	66 (41–92)	0.728
**KDIGO criteria AKI diagnosis**				
sCr ≥ 0.3 mg/dL in 48 h	2182 (68.7)	1099 (68.1)	1083 (69.4)	0.718
sCr ≥ 50% in 7 days	992 (31.3)	514 (31.9)	478 (30.6)	0.718

ICU, intensive care unit; eGFR, estimated glomerular filtration rate; CKD-EPI, The Chronic Kidney Disease Epidemiology Collaboration; AKI, acute kidney injury; sCr, serum creatinine (conversion factors for units: mg/dL to μmol/L, x88.4); KDIGO, Kidney Disease: Improving Global Outcomes.

**Table 2:** Comparison of clinical outcomes between the pre-alert and post-alert groups.

	Overall	Pre-Alert	Post-Alert	*p*
	N = 3174	N = 1613	N = 1561	
**KDIGO at diagnosis**				
KDIGO 1	2357 (74.3)	1185 (73.5)	1172 (75.1)	0.430
KDIGO 2	399 (12.6)	204 (12.6)	195 (12.5)	0.929
KDIGO 3	418 (13.2)	224 (13.9)	194 (12.4)	0.364
**Maximum KDIGO**				
KDIGO 1	1697 (53.5)	822 (51.0)	875 (56.1)	0.026
KDIGO 2	472 (14.9)	254 (15.7)	218 (14.0)	0.306
KDIGO 3	1005 (31.7)	537 (33.3)	468 (30.0)	0.117
**AKI progression**	813 (25.6)	439 (27.2)	374 (24.0)	0.117
**Nephrology consultation**	832 (26.2)	440 (27.3)	392 (25.1)	0.306
**Time from diagnosis to nephrology consultation (days)**	1 (−1 a 2)	1 (−1 a 3)	0 (−1 a 2)	0.117
**RRT**	476 (15.0)	241 (14.9)	235 (15.1)	0.929
**Length of stay (days)**	13 (7–23)	13 (7–23)	13 (7–22)	0.675
**30-day hospital readmission**	350 (18.1)	168 (18.2)	182 (18.0)	0.929
**30-day mortality**	1068 (33.6)	592 (36.7)	476 (30.5)	0.012

KDIGO, Kidney Disease: Improving Global Outcomes; RRT, renal replacement therapy.

**Table 3:** Adherence to the care bundle in the post-alert group.

	24 h before Alert	24 h after Alert	*p*
**Complete fluid chart**	1042 (66.8)	1005 (64.4)	0.073
**Creatinine request**	1366 (87.5)	672 (43.0)	0.002
**Capillary glycemia test**	1054 (67.5)	1097 (70.3)	0.039
**Glycemia >180 mg/dL**	440 (28.2)	492 (31.5)	0.024
**Hypotension**	473 (30.3)	416 (26.6)	0.029
**NSAIDs**	62 (4.0)	41 (2.6)	0.002
**Furosemide**	833 (53.4)	852 (54.6)	0.132
**ACEis/ARBs**	538 (34.5)	444 (28.4)	0.002

NSAIDs, nonsteroidal anti-inflammatory drugs; ACEis, angiotensin-converting enzyme inhibitors; ARBs, angiotensin receptor blockers.

**Table 4:** Cox univariate and multivariate proportional hazard models: 30-day mortality.

	Univariate			Multivariate		
	HR	95% CI	*p*	HR	95% CI	*p*
**Male gender**	1.02	0.90–1.15	0.847			
**Age**						
18–<40 years	Reference	Reference	Reference			
40–<65 years	1.51	1.15–1.99	0.007	1.33	1.0–1.75	0.047
65–<75 years	2.06	1.56–2.71	0.004	1.60	1.19–2.14	0.002
≥75 years	2.85	2.18–3.74	0.004	2.14	1.60–2.87	<0.001
**ICU admission**	1.25	1.09–1.45	0.005	1.24	1.07–1.43	0.004
**Baseline eGFR CKD-EPI (each 10 mL/min/1.73 m^2^)**	0.92	0.91–0.94	0.004	0.95	0.93–0.97	<0.001
**Cardiovascular disease**	1.06	0.94–1.20	0.449			
**Pulmonary disease**	1.42	1.17–1.73	0.004	1.25	1.03–1.53	0.026
**Gastrointestinal disease**	0.86	0.69–1.07	0.253			
**Neurologic disease**	1.27	1.09–1.48	0.005	1.14	0.98–1.34	0.094
**Rheumatologic disease**	0.87	0.59–1.28	0.597			
**Malignancy**	1.16	0.99–1.36	0.098	1.29	1.10–1.51	0.002
**Immunosuppressive**	0.63	0.45–0.89	0.016	0.77	0.54–1.09	0.137
**Endocrine disease**	1.02	0.90–1.16	0.847			
**Electronic AKI alert**	0.84	0.75–0.95	0.012	0.87	0.77–0.98	0.023

ICU, intensive care unit; eGFR, estimated glomerular rate; CKD-EPI, The Chronic Kidney Disease Epidemiology Collaboration.

**Figure A1:**
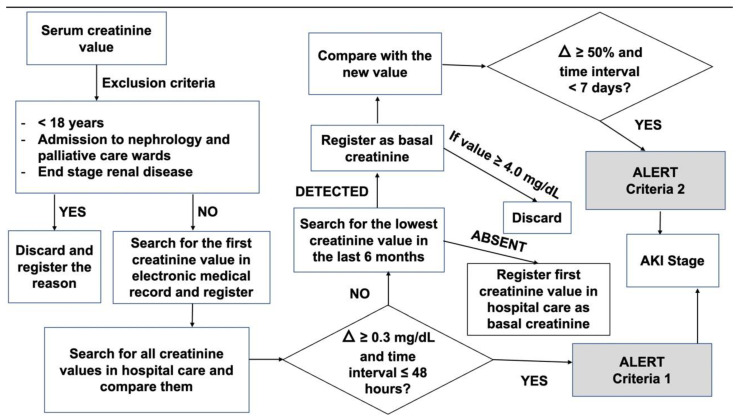
Flowchart detailing the electronic AKI alert code.

**Figure A2:**
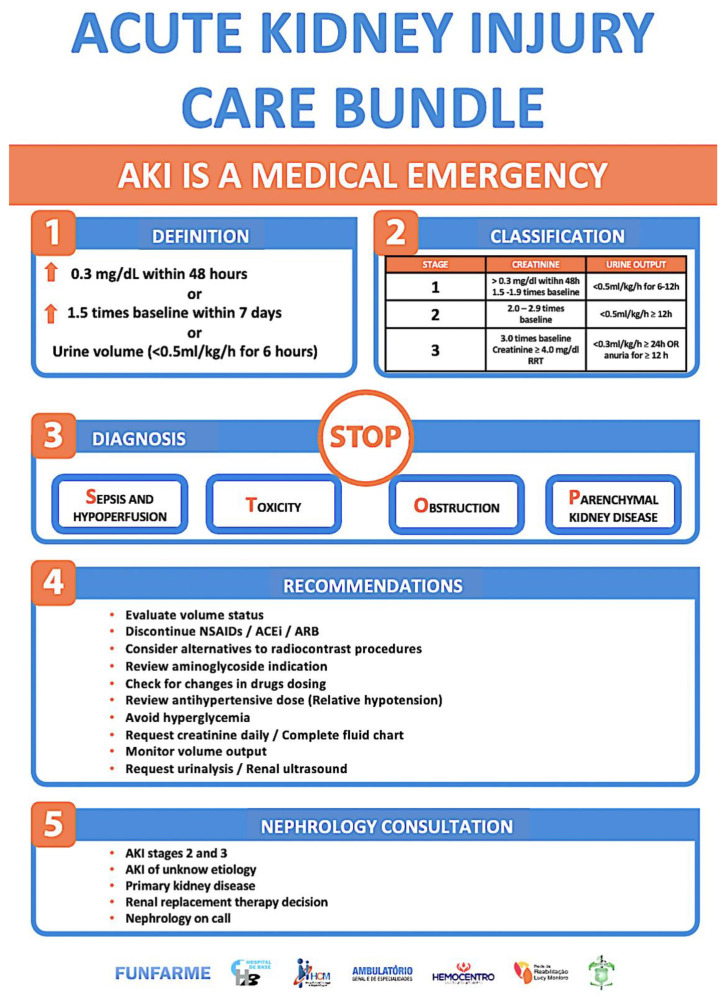
Suggested AKI care bundle.

## Data Availability

Data supporting the reported results can be obtained on request.
